# Non-invasive quantification of cardiac stroke volume in the edible crab *Cancer pagurus*

**DOI:** 10.1186/s12983-019-0344-7

**Published:** 2019-12-12

**Authors:** Bastian Maus, Sebastian Gutsfeld, Hans-Otto Pörtner, Christian Bock

**Affiliations:** 1 Alfred-Wegener-Institute, Helmholtz Centre for Polar and Marine Research, Integrative Ecophysiology, Am Handelshafen, 12 27570 Bremerhaven, Germany; 2 Department of Biology and Chemistry, University of Bremen, Bibliothekstraße 1, 28359 Bremen, Germany

**Keywords:** Crustacea, Photoplethysmography, Cardiac MRI, Stroke volume, Heart rate, Ejection fraction

## Abstract

**Background:**

Brachyuran crabs can effectively modulate cardiac stroke volume independently of heart rate in response to abiotic drivers. Non-invasive techniques can help to improve the understanding of cardiac performance parameters of these animals. This study demonstrates the in vivo quantification of cardiac performance parameters through magnetic resonance imaging (MRI) on the edible crab *Cancer pagurus*. Furthermore, the suitability of signal integrals of infra-red photoplethysmographs as a qualitative tool is assessed under severe hypoxia.

**Results:**

Multi-slice self-gated cardiac cinematic (CINE) MRI revealed the structure and motion of the ventricle to quantify heart rates, end-diastolic volume, end-systolic volume, stroke volume and ejection fraction. CINE MRI showed that stroke volumes increased under hypoxia because of a reduction of end-systolic volumes at constant end-diastolic volumes. Plethysmograph recordings allowed for automated heart rate measurements but determination of a qualitative stroke volume proxy strongly depended on the position of the sensor on the animal. Both techniques revealed a doubling in stroke volumes after 6 h under severe hypoxia (water *P*O_2_ = 15% air saturation).

**Conclusions:**

MRI has allowed for detailed descriptions of cardiac performance in intact animals under hypoxia. The temporal resolution of quantitative non-invasive CINE MRI is limited but should encourage further refining. The stroke volume proxy based on plethysmograph recordings is feasible to complement other cardiac measurements over time. The presented methods allow for non-destructive in vivo determinations of multiple cardiac performance parameters, with the possibility to study neuro-hormonal or environmental effects on decapod cardio physiology.

## Background

Among invertebrate species, brachyuran crustaceans are one of the most thoroughly studied groups, concerning their responses and vulnerability to future climate change [1]. Their importance at a global level is characterized by their abundance in benthic ecosystems, as well as the high invasive potential and the economic value of some species. As omnivorous predators and scavengers, they are potential threats to native ecosystems [2]. Commercial fisheries may be harmed or benefit from invasive crustaceans, as illustrated by Hänfling et al. [3].

The invasive potential of brachyuran crabs is supported by their capacity to tolerate changes in abiotic variables [4]. For example the thermal tolerance of crustacea was shown to relate closely to the capacities of their cardiovascular system [4, 5]. Cardiac performance parameters have been subject of physiological studies for decades. Brachyura have a degree of vascularization that is high for an invertebrate group. Heart rate (HR), stroke volume (SV), blood flow and oxygen consumption rates show periodic fluctuations in undisturbed crabs under control conditions [6–9]. It is speculated that these periodic fluctuations in cardiovascular activity may conserve energy over time [10]. Crustaceans are able to adjust their cardiac output through independently modulating HR and SV [11]. This is evidenced by constant stroke volumes with increasing heart rates above certain, species-specific temperature thresholds [12, 13]. In addition, variable haemolymph flow velocities in the sternal artery at stable heart rates under different seawater bicarbonate levels were attributed to changes in SV [14]. To understand how cardiac output as a representative of net cardiovascular performance is modulated, it is imperative to follow changes in SV and HR in otherwise undisturbed animals. Non-invasive techniques will help understand the interplay of multiple cardiac parameters in shaping cardiac and thus whole-animal performance.

Non-invasive studies of the crustacean circulatory system are complicated by its structure. The neurogenic myocardium is suspended in the pericardial sinus by elastic ligaments aiding diastolic extension of the ventricle. The ventricle has a single chamber, which is structured into a complex cavitary system by muscular walls [15, 16]. The oxygenated haemolymph is delivered from the heart through five arterial systems (for morphological overviews, see [15–18]). To adjust stroke volume, the volume of the pericardial sinus or the contractile force of the ventricle can be controlled via neuronal or hormonal action. Especially hormonal effects are long-lasting and supposedly involved in setting enhanced cardiac activities after handling or surgical procedures [19].

While mostly employed in traditional pre-clinical studies, non-invasive imaging techniques such as magnetic resonance imaging (MRI) are now applied to non-model species with high ecological importance [16, 20]: In vivo MRI has been used to study cardiovascular responses of crustaceans to decreasing temperatures and ocean acidification [14, 21]. In (pre-)clinical research, end-systolic (ESV) and end-diastolic volumes (EDV) are conventionally calculated from multi-slice cinematic (CINE) MRI scans covering the ventricle. Changes in single-slice volumes during systole and diastole allow for the calculation of the total SV and ejection fraction (EF) over one cardiac cycle. In addition to stroke volume alone, ejection fraction is a measure for the efficiency of contractile work. Lacking direct observations of ESV and EDV of crustacea in vivo, EF has not been determined thus far.

Technological advances have partly reduced the need for invasive methods to study the cardiovascular system of decapoda: HR is now commonly measured through infra-red photoplethysmographs (IR-PPG) [22], laser Doppler [23], or ultrasonic Doppler sensors [6]. These approaches are feasible because the ventricle is located dorsally, just underneath the carapace. The combined measurements of arterial haemolymph flow and HR via ultrasonic Doppler sensors have been used to calculate SV, but flow measurements in the sternal artery require surgical implantation of a Doppler probe close to the vessel [6]. Other techniques, i.e. based on thermodilution or the Fick principle (for a comparison see Burnett et al. [24]), require even more severe surgeries or suffer from a low temporal resolution, respectively. Despite reports on the qualitative determination of a stroke volume proxy (SVP) through integration of the IR-PPG signal reflected by the contracting heart [12], a validation of this concept in crustacea is still missing. The classical determination of SV, as the difference between end-diastolic and end-systolic volumes should only be possible with MRI. Measurements of these volumes in intact animals can help understand the functional processes that shape cardiac performance in response to e.g. environmental drivers.

The present study presents in vivo quantifications of cardiac performance parameters beyond HR and SV, incorporating measurements of EDV, ESV and EF for the first time in a marine crustacean. Quantitative data obtained with MRI were further used to assess the applicability of IR-PPG signal peak integrals as a proxy for stroke volume changes (SVP). Experiments were performed on the edible crab *Cancer pagurus* (Crustacea, Brachyura, Cancridae [25]). To test the methods for accuracy in the determination of SV changes, animals were subjected to severe hypoxia below 20% air saturation. In *Cancer magister,* this level of hypoxia is reported to induce a doubling in SV [26]. The opportunities of the presented methods for ecophysiological studies are discussed, as well as their advantages and limitations.

## Results

### MR imaging

**Fig. 1:**
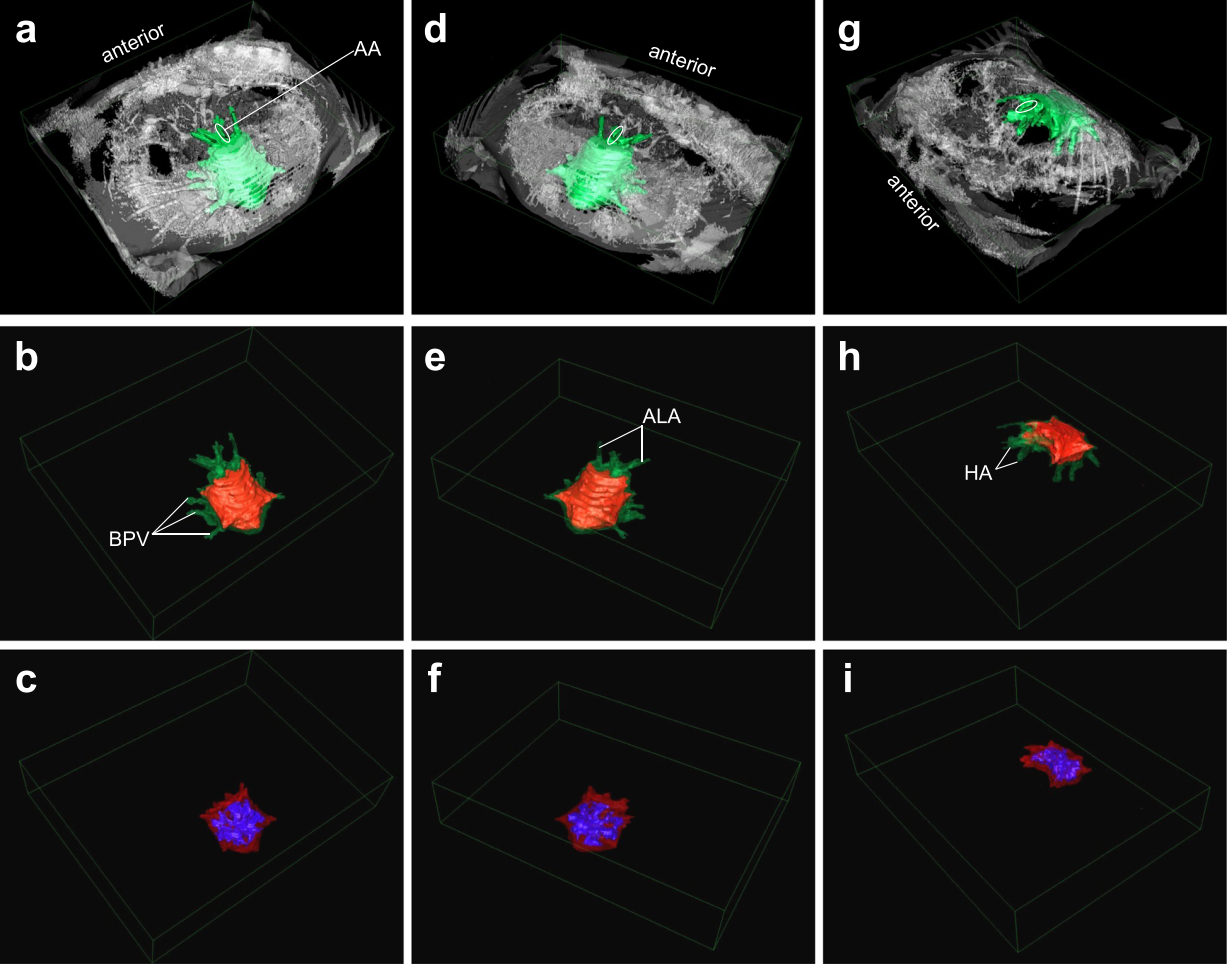
3D surface projections of pericardial sinus, myocardium and ventricular cavities in the heart of *C. pagurus*. Reconstructions are based on a stack of 14 coronal 2D slices. Columns (**a-c**; **d**-**f**; **g-i**) represent the same perspective on the structures. For easier separation, pixel intensities for body (white), pericardial sinus (green), ventricle (red) and ventricular cavities (blue) were adjusted, depending on their outline in the 2D slices. Volumes of these structures are given in the text. Dimensions of the green box in mm: 100 × 50 × 14 (*l × w × h*). Arterial and venous structures are labeled as follows: *AA* anterior artery; *ALA* anterolateral arteries; *HA* hepatic arteries; *BPV* branchiopericardial veins

**Fig. 2:**
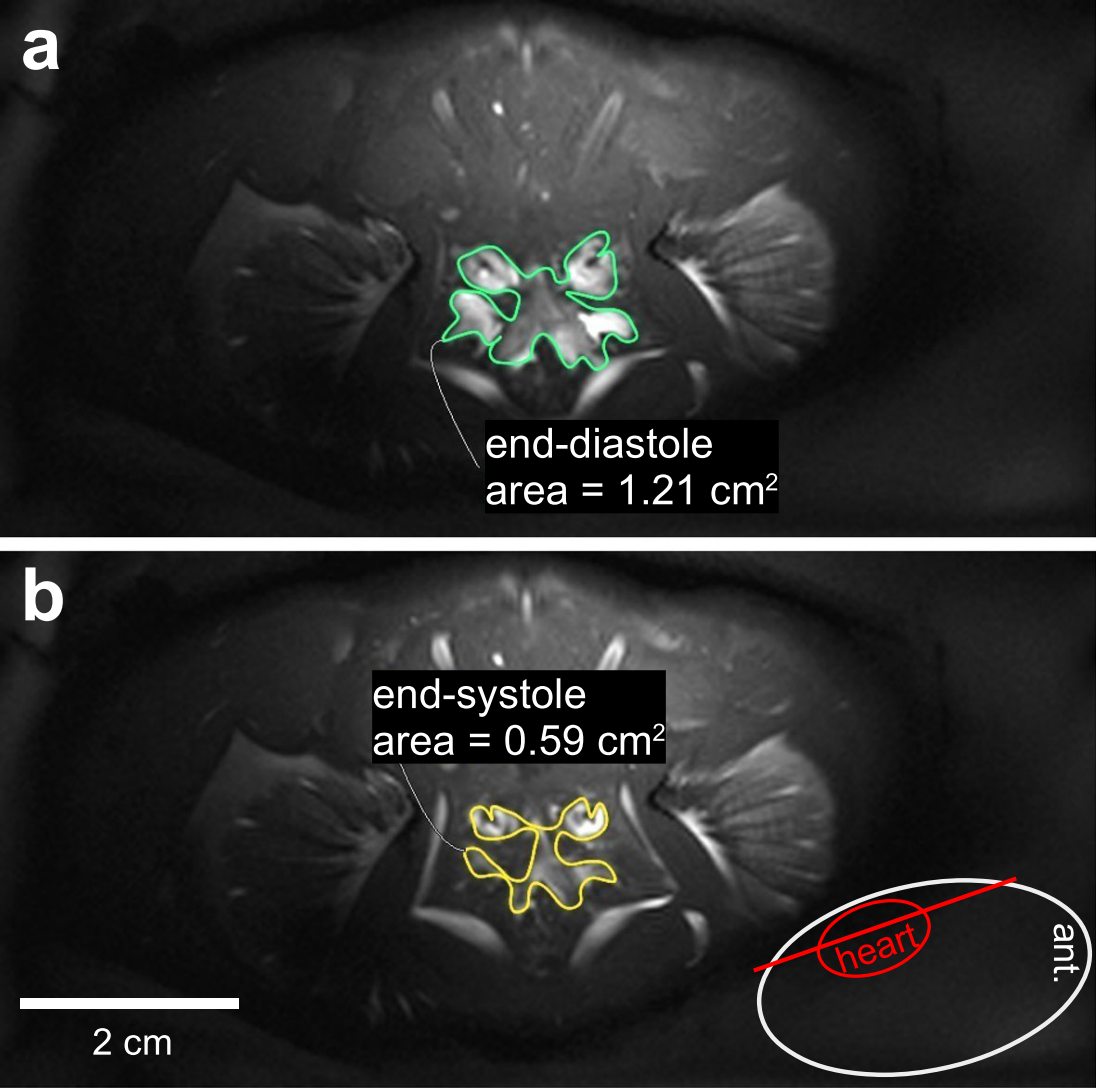
Examples of slice position for IntraGate© CINE MRI. **a**) End-diastolic and **b**) end-systolic frames from one IntraGate© CINE MRI scan. The lumen of the heart is outlined. The volumes are a) = 121 μL and b) = 59 μL. The slice position of the scan is depicted by the red line from a lateral view (anterior facing right)

Combining gap-less 2D single-slice anatomical MRI scans of the heart into a volumetric stack allowed for reconstructions of 3D surface renders of pericardial sinus, myocardium and ventricular cavities (Fig. 1). From these models, the complex, chambered inner structure of the myocardium can be studied (Fig. 2). The haemolymph-filled cavities within the ventricle are not separated by valves and are formed by myocardial folds. The largest sub-structure connects the ostia and reaches down to the sternal and posterior arteries. It covers approximately two thirds of the total inner heart volume. Three smaller cavities form at the bases of the hepatic and antero-lateral arteries and of the anterior aorta. Reconstructions of the pericardial sinus also include afferent (branchiopericardial veins) and efferent (arteries) haemolymph vessels (Fig. 1). Only the posterior artery is missing in the reconstructions, because of its small diameter [16]. The 3D surface rendered images revealed a total volume of the pericardial sinus of about 4.25 mL and a total heart volume (including ventricular cavities) of 2.52 mL. The volume of the cardiac muscle alone was 1.83 mL. The total volume of the cavities amounted to 0.69 mL in an animal with 12.5 cm carapace width, weighing 308 g. As an example, the slice positions of self-gated CINE MRI relative to the animal are shown in Fig. 2. A multi-slice CINE MRI scan revealed a stroke volume of 0.25 mL and an ejection fraction of 27.8% in this animal.

### Cine MRI

**Fig. 3:**
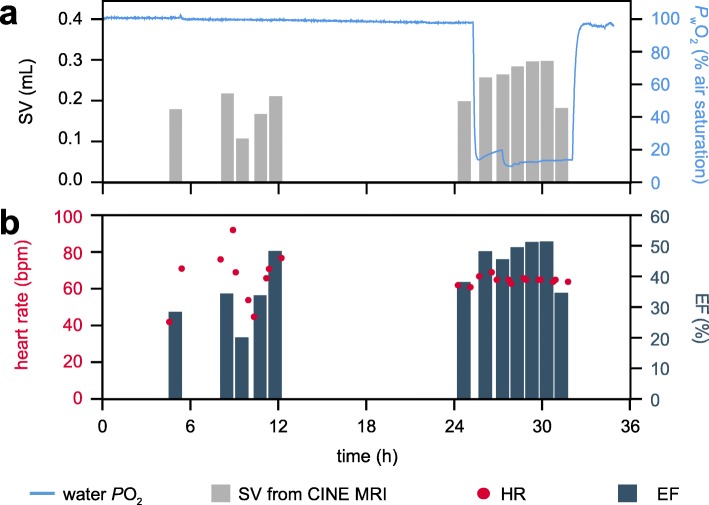
Stroke volume, heart rate and ejection fraction of *C. pagurus* at different water *P*O_2_ determined by MRI. **a**) Stroke volume (SV) and water *P*O_2_. **b**) ejection fraction (EF) and heart rate (HR). The width of the bars represents the scan time (50 min). Heart rates are shown for the first and last slice of a multi-slice IntraGate© CINE MRI scan

Self-gated cardiac CINE MRI was performed on two animals to quantify SV, EF and HR in response to severe hypoxia. Cardiac stroke volumes could be determined in a range of 0.1 mL to 0.3 mL (Fig. 3) without image distortions due to movements despite the rather long acquisition time of around 50 min for the determination of one SV. The variability in SV was greater under normoxia than under hypoxia but hypoxia led to a progressive, steady increase in SV. This is exemplified for animal 1 in Fig. 3. SV showed a continuous increase from 0.2 mL to 0.3 mL during hypoxic exposure (*P* < 0.05; Fig. 3a; Table 1). After a 5 h increase, SV declined during the last hypoxic hour. For animal 2, CINE MRI determined a SV around 0.1 mL at the start of the acute change in water *P*O_2_ that doubled to a maximum value of only 0.2 mL after 6 h of hypoxia. However, no significant change in mean SV was observed compared to control conditions, under which the SV fluctuated from 0.1–0.2 mL.

**Table 1:** Cardiovascular performance parameters of *C. pagurus* under different water *P*O_2_ acquired with CINE MRI

Condition	Normoxia		Hypoxia	
Animal	1	2	1	2
*P*_w_O_2_ during MRI (% air saturation)	97.96 ± 0.27	100.59 ± 0.24	13.20 ± 0.55*	15.04 ± 0.11*
HR from MRI (bpm)	65.50 ± 14.00	35.20 ± 17.08	64.62 ± 0.92	30.50 ± 1.52
EDV (mL)	0.506 ± 0.077	0.659 ± 0.170	0.576 ± 0.002	0.500 ± 0.020
ESV (mL)	0.398 ± 0.141	0.659 ± 0.063	0.283 ± 0.005	0.481 ± 0.141
SV from IG MRI (mL)	0.180 ± 0.040	0.177 ± 0.034	0.292 ± 0.007*	0.159 ± 0.044
EF (%)	33.87 ± 9.44	27.47 ± 4.19	50.74 ± 1.03*	23.89 ± 4.60

Values are given as means ± standard deviation. Normoxic conditions for MRI cover 24 h before the switch to hypoxia, to account for the lower number of sampling points. Hypoxic conditions reflect conditions for the last 3.5 h of low water *P*O_2_ levels. Asterisks show that the value under hypoxia is significantly different from the value under normoxia (Mann-Whitney-rank-sum-test, *P* < 0.05)

The trends in SV determined via CINE MRI are paralleled by changes in ejection fraction (Fig. 3b). Hypoxia led to a significant 1.5-fold increase in EF for animal 1. The increased EF and SV are caused by reduced end-systolic volumes at stable end-diastolic volumes (Table 1). During MR experiments, HR was determined from the navigator signals of the self-gated CINE routine. HR scattered between 40 and 95 bpm for animal 1 (Fig. 3b; Table 1) but the scatter was less for animal 2 (Table 1). In response to hypoxia, the HR variability was reduced for both animals, and a significant reduction in mean HR was found for animal 2 (Table 1).

### Infra-red photoplethysmography

**Fig. 4:**
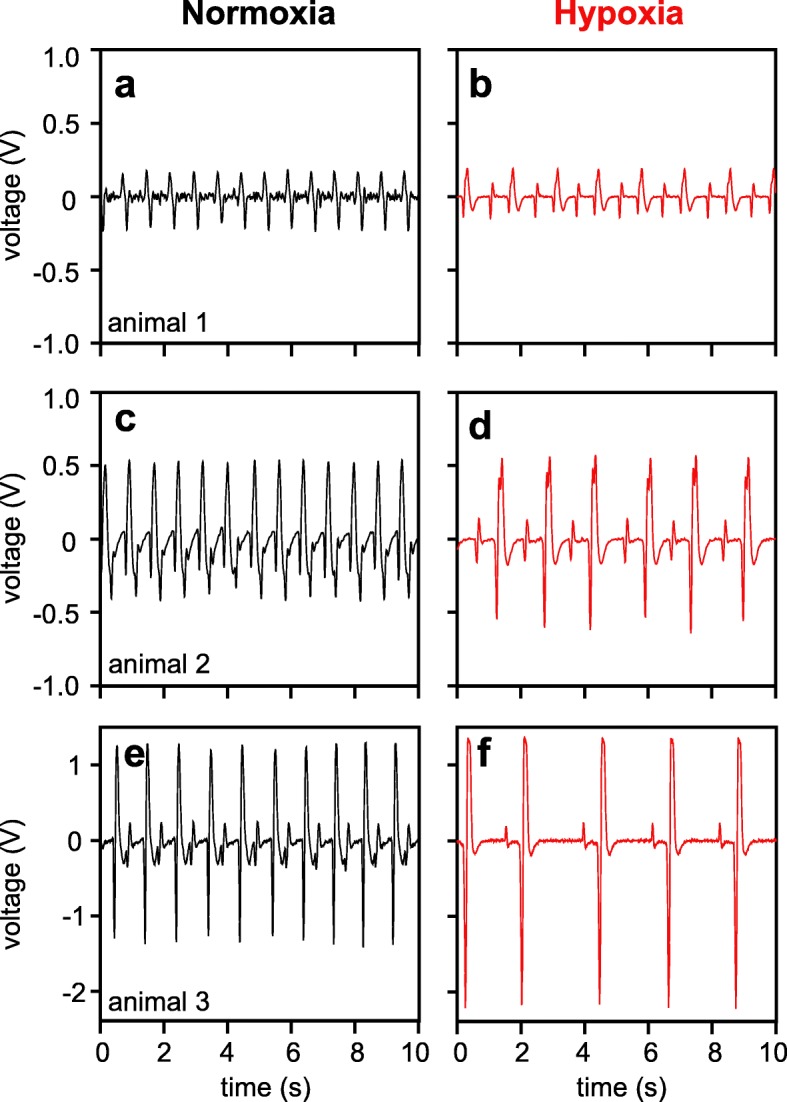
Time courses of heart beat signals of *C. pagurus* recorded with infra-red photo-plethysmography. Exemplary presentation of individual IR-PPG recordings between normoxia and hypoxia for three individuals. **a**-**b**) animal 1; **c**-**d**) animal 2; **e**-**f**) animal 3

The IR-PPG sensor was attached to the cardiac region of the carapace and its position was adjusted so periodic peaks were clearly identifiable. Still, the shape and amplitude of single peaks differed between animals and even for repeated measurements on one animal (Fig. 4). Contractions in such a recording contain two phases: The first and more prominent one is a sequence of a negative and positive deflection. This is followed by a negative overshoot before baseline values are achieved. The second phase displays a similar sequence of a negative and a positive peak, but with a much lower amplitude and shorter duration. Hypoxia usually increased the negative and positive deflections of the first phase of the cardiac cycle, and especially the negative deflection could increase in amplitude by ~ 1 V (Fig. 4e, f).

**Fig. 5:**
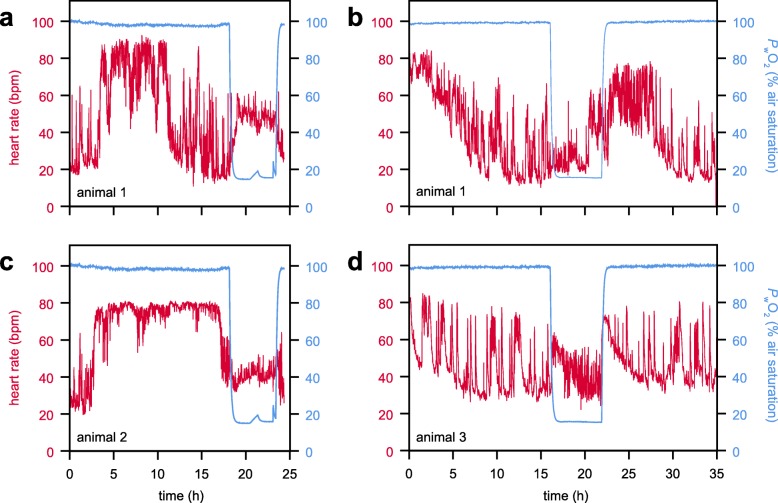
Time courses of heart rate of *C. pagurus* under different levels of water *P*O_2_. Heart rates were recorded with IR photo-plethysmographs at 12 °C water temperature. After at least 15 h of normoxic control, water *P*O_2_ was reduced to 15% air saturation by adding N_2_ gas. Hypoxic conditions were maintained for 6 h, after which the aeration was switched back to ambient air. **a** and **b** are two runs on animal 1, separated by one week of recovery; **c** = animal 2; **d** = animal 3. Note, the different/variable pattern under normoxic conditions, in contrast to hypoxia

After raw signal smoothing, noise filtering and auto-leveling to adjust different peak heights, positive peaks of the IR-PPG recordings could be detected automatically. Heart rates were found to vary between 10 and 90 beats per minute (bpm) under normoxic control conditions at 12 °C (Fig. 5). Hypoxic HR varied less in all experimental animals (i.e. reduced standard variation in Table 2), similar to the patterns found in the MRI experiments. In case of stable normoxic HR, hypoxic exposure significantly reduced mean HR by 50% (animals 2 and 4, Table 2). A maximum normoxic HR of 80–90 bpm was found in all animals (Fig. 5). Compared to this maximum, hypoxia caused a bradycardia to values between 20 and 60 bpm. Still, lowest HR for animal 1 were not found under hypoxia but under control conditions (Fig. 5a, b) and this is similar to the MRI results (Fig. 3). The return to normoxic conditions elevated HR to their normoxic maxima (80 bpm), followed by a steady decline during the next 6 h to control values (Fig. 5b, d).

**Table 2:** Cardiovascular performance parameters of *C. pagurus* under different water *P*O_2_ acquired with infra-red photoplethysmography

Condition	Normoxia				Hypoxia			
Animal	1	2	3	4	1	2	3	4
*P*_w_O_2_ during IR-PPG (%air saturation)	97.93 ± 0.36	97.93 ± 0.36	99.32 ± 0.22	97.93 ± 0.36	16.58 ± 1.64*	16.58 ± 1.64*	15.63 ± 0.12*	16.58 ± 1.64*
	99.32 ± 0.22				15.63 ± 0.12*			
HR (bpm)	31.15 ± 15.51	72.31 ± 12.12	40.99 ± 12.75	67.74 ± 1.49	47.80 ± 4.02*	41.55 ± 3.52*	37.41 ± 7.52*	33.76 ± 5.30*
	25.17 ± 13.30				35.03 ± 13.89*			
SVP from IR-PPG (log_2_ relative to normoxia)	−0.14 ± 0.57	− 0.05 ± 0.38	−0.01 ± 0.19	−0.01 ± 0.20	−0.69 ± 0.21*	0.92 ± 0.26*	0.48 ± 0.03*	−0.57 ± 0.73*
	−0.12 ± 0.67				−0.05 ± 0.34			

Values are given as means ± standard deviation. Normoxic conditions for IR-PPG cover 6 h before the switch to hypoxia. Hypoxic conditions reflect conditions for the last 3 h of low water *P*O_2_ levels. Animal 1 was subjected to IR-PPG measurements twice, separated by one week of recovery. Negative values for the SVP show reductions below average control levels. Asterisks show that the value under hypoxia is significantly different from the value under normoxia (Mann-Whitney-rank-sum-test, *P* < 0.05)

**Fig. 6:**
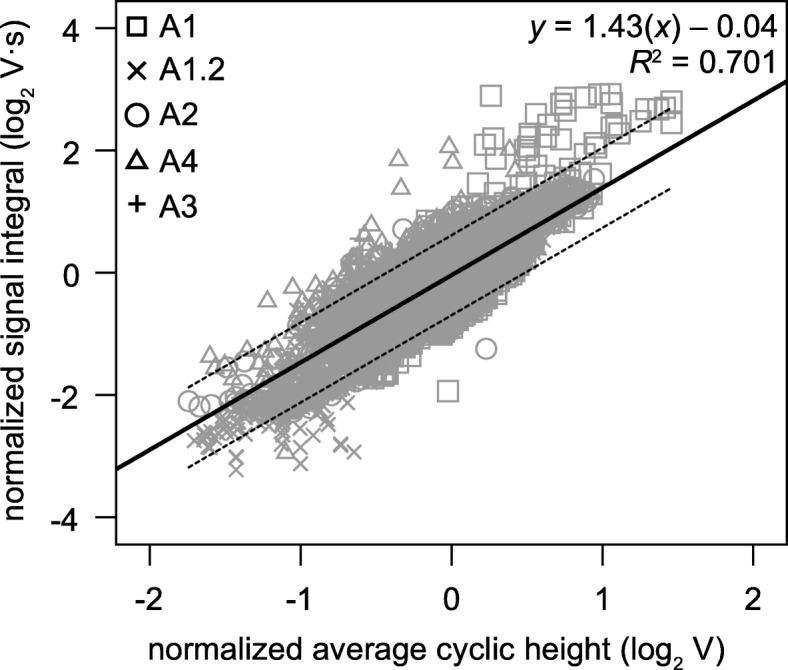
Linear correlation between average cyclic height of the IR-PPG signal and signal integral per minute. The figure includes the entire data set recorded per animal. Both parameters have been transformed to a log_2_ scale (log-log transformation). Different symbols denote different animals (A1.2 is the second run performed on animal 1). The dashed lines show the 95% confidence interval. Correlation analysis confirmed a significant positive linear correlation between the two parameters (*P* < 0.05; Pearson’s correlation coefficient 0.837)

**Fig. 7:**
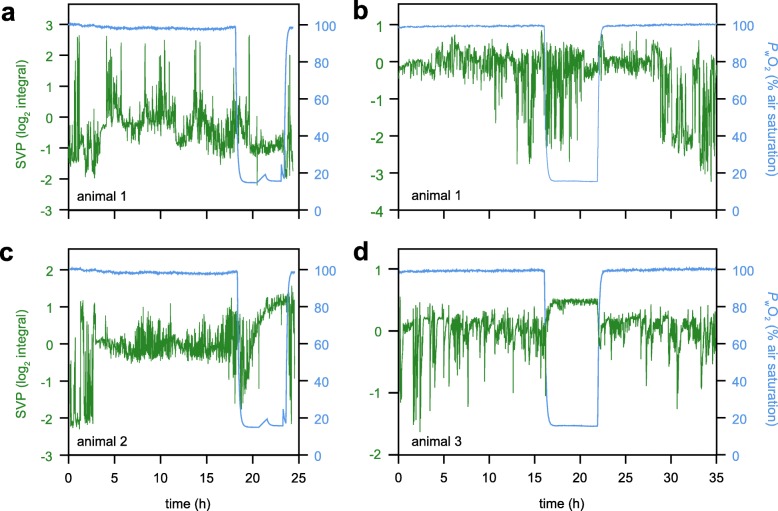
Stroke volume proxy of *C. pagurus* over time under different levels of water *P*O_2_. The cardiac stroke volume was approximated from signal peak integrals of IR-PPG recordings (cf. Figure 4) at 12 °C water temperature. Values were averaged over the last 6 h before switching to hypoxic conditions to represent controls and then transformed to a log_2_ scale. After at least 15 h of normoxic control, water *P*O_2_ was reduced to 15% air saturation by adding N_2_ gas. Hypoxic conditions were maintained for 6 h, after which the aeration was returned to ambient air. **a** and **b** are two runs on animal 1, separated by one week of recovery; **c** = animal 2; **d** = animal 3

A stronger cardiac contraction constitutes a stronger negative deflection of the IR-PPG signal. As a first step, cardiac motion was determined as cyclic amplitude per heartbeat, averaged in one-minute-intervals. The results correlate significantly with the signal integral per minute, thus showing that signal integrals are linearly affected by changes in cardiac stroke volume (Fig. 6). Generally, phases of constant heart rates are paralleled by stable signal integrals (stroke volume proxy, SVP) under normoxia (compare Fig. 5c and Fig. 7c). Animals with relatively stable HR and SVP under normoxia displayed a steady increase in SVP once water *P*O_2_ declined (Fig. 7c). Again, this is mainly caused by a larger negative deflection of the initial phase of the cardiac cycle (Fig. 4). This increase eventually leveled off after prolonged hypoxia. The significant increase in SVP was two-fold after 6 h of hypoxia for animal 2 (Fig. 7c, Table 2) but only 1.4-fold for animal 3 (Fig. 7d). Animals 1 and 4 showed no distinct changes in the SVP (Table 2; exemplified for animal 1 in Fig. 7). In contrast to heart rates, signal integrals almost immediately returned to control values, after water *P*O_2_ returned to 100% air saturation.

## Discussion

Fast and accurate SV measurements can ideally complement measurements of heart rates and can then provide a better understanding of the cardiac performance capacity of brachyuran crabs in response to abiotic drivers. Non-invasive techniques improve the value of long-term ecophysiological studies, where whole-animal performance over time (workload) is linked to e.g. climate-driven limitations [27]. They also allow for repeated measurements, to follow adaptation in an individual. In case of brachyuran crabs with their rather flexible cardio-vascular performance [9], a single cardiac performance parameter like heart rate cannot fully characterize cardiac performance as indicator for environmental tolerance thresholds [28]. This is felt important as the cardio-circulatory system is hypothesized to play a key role in thermal tolerance [29].

Using implanted Doppler flowmeters, previous studies showed heart rate and cardiac stroke volume can act independently of each other to adjust cardiac output in crustacea under exercise or environmental hypoxia [9, 26]. All non-invasive techniques applied in this study could detect the proposed doubling of SV when water oxygen levels were reduced to 15% air saturation, but when looking into detail, animals showed some important differences in cardiac performance. Cardiac MRI was able to determine these changes, despite a scan time of 50 min. This was possible, because the hypoxia-induced changes in SV were steady. To follow more dynamic SV changes, we tested the applicability of IR-PPG signal integrals, previously reported as a proxy for stroke volume in *Carcinus maenas* [12]. The different approaches to non-invasive SV measurements certainly have specific advantages and drawbacks that shall be discussed in more detail.

### Cardiovascular MRI

Static anatomical MR images allowed for the determination of the volume of the crustacean cardiac muscle and its embedded cavities at a precision of ±5 μL, given by the image resolution and slice thickness. The 3D reconstructions now allow for a detailed analysis of structure and function of the cardiac muscle during contraction and extension. The high resolution of the anatomical MR images map the complexity of the inner structure of the ventricle in vivo, complementing older morphological drawings [30]. Volumetric measurements of the heart of a decapod crustacean are certainly made difficult by these inner structures. While not strictly divided into compartments, any function of the heart’s inner structure can only be determined in vivo. Myocardial folds were identified in both, motion-free anatomic MRI and CINE MRI. They may assist in the distribution of haemolymph, in conjunction with arterial resistance and cardiac valves, but this remains to be verified.

**Fig. 8:**
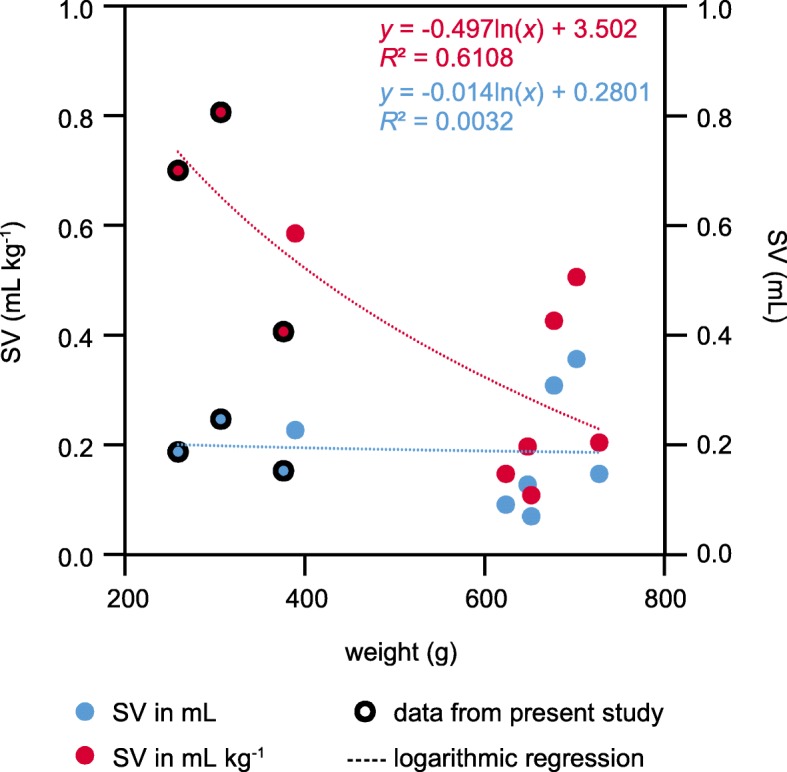
Correlation between stroke volume and body weight in brachyuran crabs. Stroke volume is given in mL and mL kg^− 1^. Data from three individuals from the present study is compared to literature data for *C. magister* (Doppler flowmeters [9, 13, 26, 31–33]) and for *C. pagurus* (Fick principle [8]). Mean SV given in these studies was divided by mean weight and fit with a logarithmic regression

The value of non-destructive CINE MRI is demonstrated by the first-ever in vivo quantification of end-diastolic volume (EDV), end-systolic volume (ESV), stroke volume (SV) and ejection fraction (EF) of a decapod crustacean. Control SVs were similar to values determined via the Fick principle [8] or Doppler flowmeters in the cognate species *C. magister* [26]. Combining present data with literature references at 12 °C [8, 9, 13, 26, 31–33] shows that SV itself remains fairly constant as animals grow (Fig. 8). Consequently, SV in mL kg^− 1^ declines with increasing weight. General conclusions on the relationship between SV and animal size would require SV measurements under well-defined physiological conditions to compensate for the natural SV variability. The time course of the SV changes in response to hypoxia is similar to literature references [26] and the results obtained via CINE MRI match those from the IR-PPG measurements: An acute reduction in water *P*O_2_ led to progressive increases in SV during the first 3 h of hypoxia and elevated SV remained relatively stable for the subsequent 3 h of hypoxic exposure.

Our data have shown that an increase of SV and EF under hypoxia is caused by a reduced end-systolic volume at stable end-diastolic volume. With CINE MRI it is now possible to non-invasively observe functional modulations of cardiac activity as determined from semi-isolated hearts in earlier studies [19]. The inotropic effects of proctolin released from the pericardial organ are suggested to elicit the cardiac responses to hypoxia [34, 35]. It seems likely that hormonal action reduces ESV under hypoxia, given the propagating changes over time. In theory, the increase in SV could have been caused by a larger EDV. Crabs usually achieve this by increasing the volume of the pericardial sinus via stimulation of the ventral alary muscles through the cardiac ganglion. However, this is reported to occur at a much faster rate [19] and could not be confirmed by our direct measurements of the end-diastolic volume. Direct measurements of EDV, ESV and EF via CINE MRI can serve as powerful tools in future studies on the contractile work of the brachyuran heart. General function, neuro-hormonal regulation or contractile responses under environmental drivers can now be studied in vivo.

One drawback of multi-slice CINE MRI in crustaceans is the rather long acquisition time of about 50 min for an accurate SV calculation to record enough slices covering the entire myocardium. In brachyuran crabs with irregular cardiac performance over time, this might result in “mixed” SV determinations, if the stroke volume changes during the acquisition time. In a separate experiment, we identified 1–3 spontaneous peaks and pauses per hour in HR under control conditions and SV may follow this pattern, as suggested by the IR-PPG recordings. These changes might be too quick to be resolved with the present multi-slice CINE MRI technique. Hypoxia induced steady SV changes, so the low time resolution of CINE MRI only had a marginally negative effect on the accuracy of the quantitative SV changes presented here. In addition, the scan time of CINE MRI can be reduced by using ultrafast MRI sequences (e.g. spiral MRI [36]) and increasing signal-to-noise ratios of the images to better delineate the lumen of the ventricle. This can be achieved using improved hardware, like high-power amplifiers and cryo-coils in combination with contrast agents [37, 38]. This was however not feasible in our current approach. Single-slice 2D scans are faster than multi-slice scans, but geometrical conversions from 2D slices to 3D volumes to calculate EDV and ESV as implemented for preclinical animal models such as rodents are not yet available for crustaceans. The complex structure and small volume of the brachyuran heart may compromise the accuracy of this approach, but can potentially be overcome with image processing techniques such as pixel similarity region growing. Considering the acquisition time of a multi-slice CINE MRI scan, slight movements of the animals may cause overlaps or gaps between myocardial structures in different slices. This may lead to miscalculations of the actual stroke volume. High accuracy is required, since SV changes in response to hypoxia were in the range of 150–200 μL, with EF changing by about 20%.

As a less time-consuming alternative to CINE MRI, cardiac output could be determined from phase-contrast (PC) flow-weighted MRI scans through the seven arteries of the crab [39]. To cover all arteries, one or two slices should be sufficient, but preliminary experiments showed that slice positioning is rather prone to errors due to animal motion. Analogous to Doppler flowmeters, PC MRI can give insight into the haemolymph flow distribution of the animal, which was found to change in undisturbed crabs and in response to temperature changes [13, 33]. Calculation of SV from PC MRI requires simultaneous HR measurements, provided by either fast single-slice CINE MRI scans or simultaneous IR-PPG recordings, as long as sensors are shielded from high-frequency electro-magnetic fields [40]. Employing such sensors, CINE MRI can be gated and used to study in more detail how different components of the cardiac cycle make up the IR-PPG signal. This may ultimately lead to the development of appropriate conversion factors that will allow SV quantifications from IR-PPG signal integrals alone.

The IntraGate© routine is able to detect HR in a wide range of values, as it was originally developed for mice (HR ~ 600 bpm). In addition, recent technical developments have considerably improved the feasibility of MRI on small-scale models [41–43].

The crustacean cardiovascular system may respond differently to environmental triggers when animals are restrained in an experimental setup [11], which was necessary for present MRI experiments. This may impact the present results and limit their applicability. However, the freely suspended chamber inside the magnet allowed for undisturbed measurements [16]. Consequently, absolute SV changes determined by CINE MRI were matched by SVP changes recorded via IR-PPG and both results supported the well-described literature findings on the effects of hypoxia on SV [26]. Separately conducted whole-animal respirometry confirmed pausing behavior in the MRI setup. This behavior was consistent whether the animal was inside the magnet or not and is described as resting condition for large, undisturbed brachyura [44]. The present techniques are thus able to yield results similar to traditional, invasive techniques and should stimulate research to further reduce the experimental impact on animal physiology.

### Infra-red photoplethysmography

For now, CINE MR images serve as a first step to explain the shape of the corresponding IR-PPG signal as shown recently for bivalves [20]. The initial negative peak is produced by the contraction of the heart (systole), while an upward slope corresponds with the filling of the heart [20]. A larger negative deflection as seen under hypoxia can be explained by the larger contraction of the heart, resulting in a smaller ESV, as evidenced by CINE MRI. The negative deflection is usually the fastest amplitude change and its deflection is mostly identical in amplitude to the following positive peak, during which the heart expands again (diastole). The total duration from maximum negative deflection back to baseline values is up to ten times longer than from baseline to maximum negative deflection. This temporal delay and the small negative overshoot after the positive peak are most likely due to the passive relaxation of the ventricle relying on elastic fibers [45]. Based on these assumptions, the integral to the one-minute-minimum (most negative peak of all systolic phases) is a valid stroke volume proxy, because this calculation covers the entire cardiac motion. It certainly allows for the determination of relative changes. Our observations indicate that the smaller secondary peak sequence in the IR-PPG signal is not caused by the contraction of the heart, but rather the blood flowing through the *arteria sternalis*, which shows pulsatile behavior delayed from the cardiac motion [16]. To resolve this question, MRI scans with a fine-tuned temporal resolution are required, ideally simultaneous to IR-PPG recordings.

The general advantage of infra-red photoplethysmography is its technical approach. Several animals can be measured simultaneously, given perfect positioning of the sensor on the animal (see below) and employing a multi-channel A/D receiver. The sensor is easy to attach and does not require surgery. The combination of dental periphery wax and superglue proved stable over several days. IR-PPG has widely replaced invasive electrographic measurements as a reliable tool for prolonged heart rate measurements in decapod crustacea [22].

Heart rate measurements and automatic peak picking were relatively unaffected by the precise position of the sensor since rhythmic signals were easily detectable, especially after adequate post-processing. Signal amplitude and peak shapes on the other hand, seemed to strongly depend on the position of the PPG sensor relative to the heart of the animal. Signal amplitude varied between noise-levels and > 3 V (compare Figs. 4a and f). Previous studies suggested no relation of signal amplitude itself to cardiac output in spider crabs *Maja squinado* [5], most likely because of the strong effect of sensor position on signal amplitude. In the present study, however, both the total amplitude of one heart beat and the signal integral showed the expected increase in SV under hypoxia. The change was progressive and final values were reached after 3 h. Animal 3 showed a lesser but still significant increase in the SVP, compared to animal 2. The recorded relative increase might have been lower because it is possible, that the contraction of the heart was not fully covered by the sensor (Fig. 4f and 5d) – signal overflow seems to have led to a cut-off beyond a quick and stable factorial increase of 1.4. Too high signal inputs can be compensated with a reduced amplifier voltage, but weak signals could not be brought up to similar prominence. The effect of sensor positioning on signal quality may therefore limit the use of IR-PPG signal integrals as SVP. Distorted peak shapes due to placement of the sensor may explain why animal 1 rather showed a depression of SV under hypoxia (Fig. 6a). However, this animal has independently shown a quantitative increase in SV during MR experiments, arguing for measurement artifacts during IR-PPG recordings. An inaccurate placement of the sensor can be judged by the prominence of the negative deflection at the beginning of the cardiac circle (compare Fig. 4a with 4c and 4e). If this deflection is not clearly visible under control conditions (Fig. 4a), the IR-PPG signal does not qualify the integral as viable proxy for stroke volume changes.

Continuous HR recordings showed high inter- and intra-individual variability under control conditions. Our observations of variable heart rates agree with previous results obtained by various techniques [8, 9, 13, 33]. Periodically fluctuating activities of cardiac and ventilatory systems were attributed to saving energy over time and can only be sustained when oxygen availability is high [7]. Depressed HR under hypoxia were reported for *C. pagurus* [8] and *C. magister* [26], but these studies typically compare hypoxic bradycardia to stable, high control heart rates. The present data confirm this conclusion when comparing normoxic maximum HR with average hypoxic HR. Reduced oxygen levels have been demonstrated to reduce the HR of isolated hearts by depressing burst rates of the cardiac ganglion [46]. Generally, hypoxic HR displayed considerably less variation than normoxic controls. This reduced variability may be a more fundamental effect in vivo, compared to the aforementioned bradycardia. Future studies of cardiac activity in decapoda should incorporate pattern analyses for HR recordings to gain more detailed insight into performance changes over time and to complement the established effects on mean HR.

Under control water *P*O_2_, changes in HR are paralleled by changes in SVP, as was also suggested from Doppler measurements [9]. The functional link between HR and SVP then changes under severe hypoxia, with HR generally decreasing and SVP increasing. In principle, these findings support the signal integral as a proxy for stroke volume changes, at least in small individuals, given an adequate placement of the sensor covering the entire heart of the animal. Still, at best, IR-PPG can only qualitatively observe SV changes, since morphological assumptions and conversion factors are needed for quantitative analyses [47].

## Conclusions

Stroke volumes are an important parameter of the crustacean cardiovascular system allowing for the modulation of cardiac output independent from heart rates. CINE MRI presents unique opportunities to study cardiac performance in vivo: It enables direct measurements of EDV and ESV, and thus SV and EF, revealing functional properties of the heart in intact animals. Current techniques suffer from relatively low temporal resolution. Fast changes in cardiac performance can be followed by IR-PPG. Given correct positioning of the sensor on the animal, the signal integral is representative of the change in cardiac motion and thus a viable stroke volume proxy. Still, further morphological references are necessary for quantitative extrapolations in crustacea.

Non-invasive techniques become increasingly easy to use and are applied to animal models beyond their initial design. This study complements the findings from previous studies on cardiac function in brachyura and presents approaches to further improve our understanding of the interplay of different cardiovascular performance parameters and their neuronal or hormonal control. MRI and IR-PPG support repeated measurements on one animal, benefitting mechanistic studies through a high level of detail within technical limitations. Further technical refinements will allow for accurate determination of cardiac performance over time in brachyuran crabs. Both methods can already be incorporated in long-term acclimation experiments to evaluate the time-course of responses to – for example – climate drivers or neurohormonal stimulation.

## Material and methods

### Experimental animals and setup

Edible crabs *Cancer pagurus* were caught via net fishing from the research vessel Uthörn around the island of Helgoland in the North Sea between autumn 2017 and autumn 2018. Animals were transported to the aquaria of the Alfred-Wegener-Institute, Bremerhaven, Germany and kept in natural seawater at 12 °C and 32 salinity. They were fed twice a week ad libitum with frozen mussels or shrimps. Feeding was stopped at least 48 h before any experimental treatment [48]. Animal carapace width was between 11.5 and 13.4 cm, at a fresh weight of 245–377 g. One animal was used for anatomic reference scans. The effects of hypoxia on heart rates and stroke volumes were studied in four different animals. During all experiments, animals remained in constant darkness to reduce disturbance.

To study changes in cardiac SV in *C. pagurus*, animals were subjected to changing water *P*O_2_ [26]. The water for the experimental setup was aerated with ambient air for normoxic conditions (control). Severe hypoxia below the animal’s critical *P*O_2_ of 15% air saturation (*P*O_2_ ≈ 3–4 kPa [8]) was achieved through aeration with an air-nitrogen mix (PR4000 multi gas controller; MKS Instruments; Andover, MA USA). At this *P*O_2_, animals show severe drops in oxygen consumption rates and heart rates [8]. Water *P*O_2_ was monitored in all setups with a temperature-compensated oxygen optode (FIBOX 3; PreSens; Regensburg; Germany), using software PSt6 (v.7.01; PreSens) after a two-point calibration in seawater aerated with ambient air for 100% saturation or aerated with N_2_ gas for 0% oxygen saturation.

After at least 18 h of normoxic conditions, water *P*O_2_ was lowered acutely to 15% air saturation, as described above. The transition was completed within 30–45 min and hypoxia lasted for up to 6.5 h in both setups. The switch back to normoxic conditions took 30–50 min. All animals survived the experiments and were subsequently placed back in the holding aquaria.

### MR imaging

MRI experiments were conducted according to the recently published recommendations for imaging crustaceans [16]. Animals were placed in a plastic chamber (volume = 1 L) and were attached to the removable lid of the chamber with Velcro©, but were able to move their legs. The chamber was connected to a 40 L tank, supplying seawater at 12 °C and at a flow rate of 200–400 mL min^− 1^. An oxygen optode was placed directly before the chamber to record changes in water *P*O_2_, as described above. The experiments were carried out in a 9.4 T horizontal MR imaging scanner with a 30 cm bore (BioSpec 94/30 US/R Avance III; Bruker BioSpin; Ettlingen; Germany). A ^1^H volume radio-frequency (RF) resonator, with an inner diameter of 154 mm and a maximum peak power of 2 kW was used for signal excitation. A 40 mm receive-only surface RF coil was used for signal reception and was placed on top of the chamber over the cardiac region of the animal. After adjustments of hardware and magnetic field homogeneity, pilot scans in three perpendicular orientations were conducted to optimize the position of the receive coil relative to the animal’s heart (fast low-angle shot; echo time = 4 ms; repetition time = 100 ms; flip angle = 30°; 128 × 128 pixels; field of view = 120 × 120 mm^2^; slice thickness = 2 mm). The MRI scanner was operated using ParaVision v6.0.1 (Bruker BioSpin; Rheinstetten; Germany).

Animals were allowed to recover from handling stress over one night. Their cardiac performance was then studied under normoxic conditions for another 12-24 h. Performance studies were preceded by the determination of the general volumes of the myocardium, its enclosed cavities and of the pericardial sinus in one animal. Volumes were calculated from 3D reconstructions, based on a stack of consecutive coronal single-slice 2D gradient echo (fast low-angle shot; FLASH) scans, with the following parameters: TE = 9.29 ms; TR = 40 ms; 16 averages; flip angle = 30°; 500 × 375 pixels; FOV = 100 × 75 mm^2^; slice thickness = 1 mm. Image contrast was enhanced using motion averaging and RF spoiling. The gapless single-slice 2D scans were combined into one 3D stack using Horos (v3.3.2; LGPL license, horosproject.org, Nimble Co LLC d/b/a Purview, Annapolis, MD, USA). Outlines of pericardial sinus, myocardium and its cavities were manually selected in each slice. After manually assigning different pixel values to the respective structures, their volumetric extent was visualized as a 3D surface projection map.

SV was determined from multi-slice IntraGate© CINE MRI (Bruker BioSpin, Ettlingen, Germany) using a rectangular approach (midpoint rule) – a simplified version of the Simpson’s-rule-approach [49]. End-systolic (ESV) and end-diastolic volumes (EDV) of the myocardium and its enclosed cavities were calculated from 2D multi-slice self-gated IntraGateFLASH© scans. Triggering was performed by an in-slice navigator. 10 frames per cardiac circle were recorded with the following scan parameters: TE = 4.032 ms; TR = 9 ms; flip angle = 60°; 256 × 128 pixels; FOV = 100 × 50 mm^2^; 11 slices; slice thickness = 1 mm. 120 samples were recorded for every *k*-space line per slice. To enhance image contrast, scans were recorded with RF spoiling and an optimized loop structure for multi-slice acquisitions (angio mode). Preliminary trials confirmed that positioning of multi-slice IntraGate© stacks did not affect the calculated SV, as long as the entire myocardium was covered. For the data presented here, the stacks were placed parallel to the carapace arching over the heart. The scan duration was 50 min. Heart rates were quantified for each slice from the IntraGate© navigator signals with LabChart (v8.1.13; ADInstruments; Dunedin; New Zealand). Signal peaks were counted using LabChart’s finger pulse function after signal smoothing and filtering. Visual inspection confirmed optimal peak detection. Finally, IntraGate© scans were reconstructed using the mean HR for this scan. This improved the quality of the reconstruction in that cardiac motion was clearly identified. Despite variations in HR (and probably also SV) during the scan acquisition, this method resulted in the determination of mean SV. Outlines of the myocardium and the enclosed cavities were manually selected in the 4D dataset, in both end-systolic and end-diastolic frames (Horos v3.3.2; horosproject.org). The respective area per slice (*A*_ED_ or *A*_ES_ in mm^2^) was multiplied with slice thickness (SI in mm), yielding in-slice EDV and ESV. Total EDV and ESV were the sum of all slices, with *s* as slice number and *n* as maximum slice number. SV was calculated as difference between total EDV and ESV:

1 $$ \mathrm{SV}=\sum \limits_{s=1}^n{\left({A}_{\mathrm{ED}}\cdot \mathrm{SI}\right)}_s-\sum \limits_{s=1}^n{\left({A}_{\mathrm{ES}}\cdot \mathrm{SI}\right)}_s $$

Ejection fraction (EF) as measure for the efficiency of single heart beats was calculated as:

2 $$ \mathrm{EF}=\frac{\mathrm{SV}}{\mathrm{EDV}}\cdot 100\% $$

### Infra-red photo-plethysmography

Individual crabs were strapped to a plastic grid with cable ties to restrain movement (*n* = 4, with two individuals also studied with MRI). IR-PPG sensors (isiTEC; Bremerhaven; Germany) were fixed watertight to the cardiac region of their carapace with super glue and dental wax. The cardiac region is outlined by small grooves slightly posterior to the axial axis on the dorsal side of the carapace. A maximum of three animals were then placed in a 40 L tank filled with natural seawater at 12 °C.

IR-PPG signals were amplified with a 5 V amplifier and recorded with LabChart at a sampling rate of 1 k s^− 1^ (v7; ADInstruments; Dunedin; New Zealand). Similar to the IntraGate© navigators, signal maximum peaks were counted automatically, using the built-in finger pulse peak detection. To optimize the automatic routine, the raw signal was smoothed (triangular window width = 0.1–1 s) and corrected for baseline noise (median filter width = 3–10 points; high-pass filter = 0–3 Hz). To account for the variable amplitude of the peaks, auto leveling was applied (window width = 0.5–5 s). Successful peak picking was confirmed by operator control. Peaks were grouped in one-minute-intervals, resulting in heart rate as beats-per-minute (bpm). It was observed that stronger cardiac motion results in larger signal deflection and thus the height of each cardiac cycle (maximum signal – minimum signal) was calculated in LabChart and averaged for one-minute intervals. The signal peak integral as a proxy for cardiac SV was also determined at one-minute intervals with integrals calculated as the sum of the data points minus the lowest value in the selection, multiplied by the sample interval of 1 min, using the rectangular rule (see [12] for further details). The integrals were determined from the unprocessed IR-PPG signal. To account for differences in peak shape and area, average cyclic height and signal integrals are presented as relative to the mean of the last 6 h of normoxia (control). Transformation to a log_2_ scale then allows for inter-individual comparisons of proportional changes in cardiac motion and SVP in response to hypoxia.

### Statistics

To assess how well the signal integral represents changes in cardiac motion, it was correlated with the average cyclic height. The quality of a linear regression was determined by calculating Pearson’s correlation coefficient. For IR-PPG experiments, normoxic control conditions were defined as the last 6 h before switching to hypoxic aeration. For MRI experiments, normoxic control conditions are defined as the last 24 h before the hypoxic exposure, to account for the lower number of data points. The effect of hypoxia on cardiovascular performance parameters was assessed starting 3 h after 20% air saturation were reached (i.e. covering the last 3.0–3.5 h of hypoxic exposure). Within these limits, the Shapiro-Wilk test and Levene’s test confirmed non-normal distribution and unequal variance for water *P*O_2_, HR, EDV, ESV, EF and SV. Outliers within groups were identified through Grubb’s test at α = 0.05. The Mann-Whitney rank-sum test was used to identify differences between normoxic and hypoxic conditions for water *P*O_2_, HR, EDV, ESV, EF and SV in each individual. Differences were deemed significant at α = 0.05. All statistical analyses were conducted with SPSS 25 (IBM Corp.; Armonk, NY; USA). Values are given as means ± standard deviation for a specific time period.

## Data Availability

The datasets generated and analyzed for the current study are available in the PANGAEA repository, https://doi.pangaea.de/10.1594/PANGAEA.909440. MRI datasets used for this study are available from the corresponding author on request.

## Abbreviations

bpm: Beats per minute
CINE: Cinematic
EDV: End-diastolic volume
EF: Ejection fraction
ESV: End-systolic volume
FLASH: Fast low-angle shot
FOV: Field of view
HR: Heart rate
IR-PPG: Infra-red photoplethysmograph
MRI: Magnetic resonance imaging
PC: Phase contrast
*P*O_2_: Partial pressure of oxygen
RF: Radio-frequency
SV: Stroke volume
SVP: Stroke volume proxy
TE: Echo time
TR: Repetition time
